# Ivabradine drug utilization study in five European countries: A multinational, retrospective, observational study to assess effectiveness of risk‐minimization measures

**DOI:** 10.1002/pds.4880

**Published:** 2019-09-04

**Authors:** Linda Salem, Alexandre Malouvier, Jon Blatchford, Elena Rivero‐Ferrer, Nicolas Deltour, Emmanuelle Jacquot

**Affiliations:** ^1^ Department of Pharmacoepidemiology and Real World Evidence Les Laboratoires Servier CEDEX France; ^2^ PRA Health Sciences Levallois‐Perret France; ^3^ PRA Health Sciences Reading United Kingdom; ^4^ RTI Health Solutions Barcelona Spain

**Keywords:** benefit‐risk balance, chronic stable angina pectoris, drug utilization, heart rate, ivabradine hydrochloride, pharmacoepidemiology, risk‐minimization measures

## Abstract

**Purpose:**

This drug utilization study of ivabradine evaluated prescriber compliance with the new risk minimization measures (RMMs), communicated starting 2014 following preliminary results from the SIGNIFY study.

**Methods:**

This was a multinational (five European countries) chart review study with two study periods: pre‐RMM and post‐RMM. Patients initiating ivabradine for chronic stable angina pectoris in routine clinical practice were identified across general practitioners and specialists. The primary outcome analysis evaluated the compliance with the new RMMs, ie, use in patients with a heart rate greater than or equal to 70 bpm at initiation, no doses higher than those recommended in the summary of product characteristics (SmPC) at initiation and during 6 months of follow‐up, and no concomitant use of verapamil or diltiazem.

**Results:**

Overall, 711 and 506 eligible patients were included in the pre‐RMM and post‐RMM periods, respectively. The percentage of patients prescribed ivabradine according to the new RMMs increased significantly in the post‐RMM period (70.6% and 78.4% in the pre‐ and post‐RMM periods respectively; *P* value = .0035). The compliance to RMMs increased for all the criteria assessed independently: the proportions of patients with (a) heart rate ≥ 70 bpm at initiation (79.4% and 85.2%, respectively; *P* value = .0141), (b) no dose higher than the SmPC doses at initiation and during follow‐up (92.8% and 94.1%, respectively; *P* value = .3957), and (c) no concomitance with verapamil or diltiazem (96.1% and 99.2%, respectively; *P* value = .0007).

**Conclusions:**

The RMMs for ivabradine were well implemented across the five participating European countries confirming a favorable benefit‐risk balance of ivabradine in chronic stable angina pectoris.

**Key Points**

- Following the SIGNIFY study, the benefit‐risk ratio of ivabradine was reassessed in 2014, and risk minimization measures (RMM) were recommended.
- Compliance to RMM was evaluated in a drug utilization study (DUS) across five European countries.
- The study results show that the heart rate at treatment initiation, ivabradine dosing at initiation and during follow‐up, and concomitant use of verapamil or diltiazem were in line with the updated summary of product characteristics (SmPC).
- In conclusion, the RMMs were well implemented across the five participating countries confirming a favorable benefit‐risk balance of ivabradine in chronic stable angina pectoris.

## 1 INTRODUCTION

Ivabradine hydrochloride (Procoralan/Corlentor) is a selective inhibitor of the cardiac pacemaker if current, with corresponding reductions in cardiac workload and myocardial oxygen consumption.^1^ Ivabradine is indicated in Europe from 2005 for the symptomatic treatment of chronic stable angina pectoris in patients with normal sinus rhythm and a contraindication to, or intolerance of beta blockers. Extensions of the indication were subsequently approved in combination with beta‐blockers in patients inadequately controlled despite an optimal beta‐blocker dose and heart rate (HR) > 60 bpm (October 2009) and in chronic heart failure (HF) in patients with sinus rhythm and HR ≥ 75 bpm (February 2012). The recommended ivabradine starting dose for these indications was 5‐mg bid, with consideration of 2.5‐mg bid for patients aged 75 years and older. The recommended maintenance dose was 7.5‐mg bid.

The SIGNIFY study^2^ was a randomized clinical trial evaluating ivabradine at a starting dose of 7.5 mg bid (5‐mg bid if age ≥ 75 years) and a maintenance dose of 10‐mg bid in 19 102 patients with coronary artery disease without HF. Results showed an increase of cardiovascular events, possibly because of bradycardia in a subgroup of patients with angina of Canadian Cardiovascular Society Class II or higher. These findings triggered a benefit‐risk reevaluation by the European Commission^3^ in May 2014, and a direct health care professional communication (DHPC) was disseminated in Europe in June 2014 to inform prescribers and remind them of the current conditions of use of the product in patients with angina pectoris.

Ivabradine benefit‐risk ratio was reassessed by the Pharmacovigilance Risk Assessment Committee (PRAC) in November 2014 and was found to remain positive for its authorized indications.^4^ The PRAC recommended to increase the resting HR threshold of patients with angina pectoris from greater than 60 to greater than or equal to 70 bpm before treatment initiation, contraindicate concomitant use of ivabradine with verapamil or diltiazem, and reinforce current posology including initial (5‐mg bid) and maintenance (7.5‐mg bid) maximal doses. Previous information found in the summary of product characteristics (SmPCs) regarding HR monitoring and warning of use in patients with atrial fibrillation was also reinforced. As routine and additional risk minimization measures (RMMs), the SmPC was updated accordingly, and a second DHPC was distributed to inform prescribers in Europe from December 2014.

Following the PRAC recommendations, a drug utilization study (DUS) was conducted to evaluate how ivabradine is used in patients with chronic stable angina pectoris in routine clinical practice and the consistency of ivabradine prescribing with the aforementioned PRAC recommendations.

## 2 METHODS

### 2.1 Study design overview

This retrospective cohort study collected data from medical records (chart review) of patients initiating ivabradine for chronic stable angina pectoris in routine clinical practice in five European countries. Data, from start of treatment until 6 months, describing ivabradine new users' characteristics and ivabradine patterns of use were collected retrospectively from patients' charts by the physicians.

**Figure 1:**
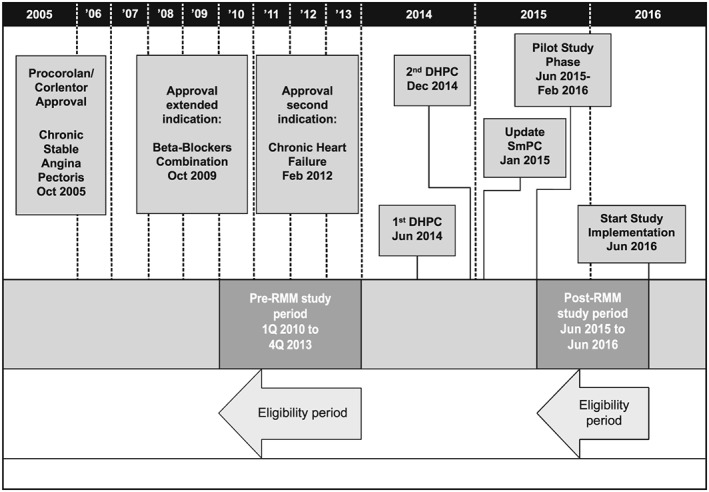
Study overview. DHPC = Direct health care professional communication; SmPC = Summary of Product Characteristics; RMM = Risk‐minimization measures

The study design included two study periods: pre‐ and post‐RMM (Figure 1):

- Pre‐RMM: before implementation of the new RMM, from January 2010 to December 2013.
- Post‐RMM: after implementation of the new RMM, from end of June 2015 to end of June 2016.

For each study period, ivabradine initiation was defined as the first date in which a patient was treated with ivabradine in the considered study period, provided that the patient had not received ivabradine during the previous 6 months.

The targeted countries were France, Germany, Italy, Spain, and the United Kingdom (UK). Countries' selection was based on ivabradine volume sales, geographic representation of the European Union (EU), and ability to represent a variety of medical practices, in term of specialty and practice settings.

A pilot study was conducted to identify potential challenges in the study design such as the shared‐care management (SCM) (ie, when patient care was shared between different physicians and not only the participating physician), to evaluate practical aspects of implementation, and test the data collection form. The protocol was registered and made publicly available on the European Medicine Agency electronic Register of Post‐Authorization Studies (EU PASS 19522).

### 2.2 Physician and patient enrollment

A total of 600 patients per study period were targeted taking into account the clustering effect and other contingencies such as missing data, allowing for an absolute precision of at least 5%. Patients were identified across a variety of physicians' specialties, including general practitioners (GPs) and specialists (cardiologists or internists) practicing in outpatient settings (private practices or hospital outpatient clinics). The physicians were targeted to allow the recruitment of patients whose treatment modalities reflected, to the extent possible, prescribing patterns in each country. The targeted patients' distribution between specialties within each country was defined according to the distribution of national sales and information on SCM obtained during the pilot study. There were 70 targeted active sites. Sampling of participating physicians was performed on IMS Health lists (Sponsor's list for France) of physicians.

The source population included all patients who had initiated ivabradine treatment for chronic stable angina in regular clinical practice in one of the study periods. The initiation could have been done in the same participating site or elsewhere, provided that key data at initiation were present in the patients' medical records. To be included, patients were required to have documented initiation of ivabradine treatment during one of the study periods, chronic stable angina as the indication for ivabradine initiation, and provision of informed consent for study participation, where applicable. Exclusion criteria included ivabradine prescribed for an indication other than chronic stable angina, documented ivabradine use in the previous 6 months, and participation in an ivabradine clinical trial simultaneously. Sites began identifying patients when all appropriate approvals from competent authorities and ethics committees were received.

To avoid a cluster effect and to ensure a sufficient number of participating sites allowing to assess different practice modalities, the physicians were informed that they could not include greater than 20 patients by period for a specialist and greater than 10 patients by period for a GP. To avoid selection bias, if a site had a larger number of patients than the maximum threshold, physicians had to organize the eligible patients in alphabetical order by surname and to start patient data abstraction in ascending or descending order whether the last number of the site ID was odd (eg, XX1) or even (eg, XX2).

### 2.3 Analysis

Primary outcome was the compliance with the composite of the four criteria in the SmPC assessed before and after RMMs implementation: (1) use in patients with a HR threshold greater than or equal to 70 bpm at initiation; (2) no doses higher than 5‐mg bid at treatment initiation; (3) no doses higher than 7.5‐mg bid during 6‐month follow‐up; and (4) no concomitant use of verapamil or diltiazem at treatment initiation or during 6‐month follow‐up. The primary analysis evaluated the proportion of patients prescribed ivabradine according to the four criteria of the SmPC.

The secondary analyses compared the demographics and specific baseline comorbidities of ivabradine new users in routine clinical practice, pre‐, and post‐RMM. Total treatment duration was defined as the time between date of treatment initiation and date of treatment discontinuation or date of censorship for patients who did not discontinue treatment during the 6‐month follow‐up.

Primary and secondary analyses were stratified by study period. Primary analyses were also performed by country and by specialty. The primary analysis was conducted in the patients' set with complete data. However, a patient who was not compliant with one of the four SmPC criteria was deemed noncompliant, regardless of the completeness of data for the other criteria. Confidence intervals (CI) and *P* values for the difference in proportions of patients satisfying each criterion were estimated using the Wilson score with continuity correction (Newcombe score^5^). For the primary outcome, sensitivity analyses were performed for missing data, based on patients' set with missing data in the denominator for proportions, and by physicians' initiator status, either the participating physician is an initiator or a subsequent prescriber, to explore the impact of SCM on the primary outcome and the missing data proportion. Analyses were conducted using SAS statistical software (SAS Institute, Cary, North Carolina), version 9.4.

## 3 RESULTS

### 3.1 Disposition

Of the 138 741 physicians in the source list with relevant contact information, 60 675 physicians were contacted. The percentage of interested physicians overall was 0.86%. Of these, 13% participated in the study. A total of 68 physicians were active (included at least one eligible patient). Active physicians' distribution by country and specialty is presented in Table 1.

**Table 1:** Total number of physicians recruited by country and specialty

Variable	France	Germany	Italy	Spain	UK	Total
No. of physicians contacted	12531	9304	21238	13300	4302	60675
GP	6996	6789	16039	10041	3167	43032
Specialists	5535	2515	5199	3259	1135	17643
No. of interested physicians (n [%])^a^	119 (0.95)	83 (0.89)	176 (0.83)	107 (0.80)	35 (0.81)	522 (0.86)
GP	52 (0.74)	45 (0.66)	67 (0.42)	63 (0.63)	25 (0.79)	252 (0.59)
Specialists	63 (1.14)	38 (1.51)	97 (1.87)	37 (1.14)	5 (0.44)	240 (1.36)
Missing	4 (0.03)	0	12 (0.06)	7 (0.05)	5 (0.12)	30 (0.05)
No. of qualified physicians with signed agreement^b^	22	26	11	15	11	85
GP	9	15	1	6	9	40
Specialists	13	11	10	9	2	45
No. of active physicians^c^	17	18	11	12	10	68
GP	7	11	1	4	8	31
Specialists	10	7	10	8	2	37

Abbreviations: GP, general practitioner; RMM, risk minimization measures; UK, United Kingdom.

a Percentages presented between parentheses are based on the number of contacted physicians within each country. Total includes two physicians with missing country.

b Physicians were qualified for activation if they had the potential to contribute the minimum number of patients treated with ivabradine in at least one study period.

c Active physicians were participating physicians who had included at least one eligible patient in the study.

**Figure 2:**
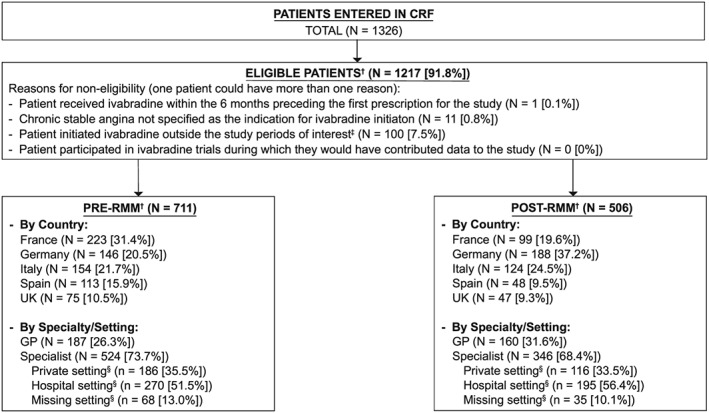
Overall patient recruitment. CRF = Case report form; GP = General Practitioner; RMM = Risk‐minimization measures; UK = United Kingdom. ^†^Eligible patients are those who satisfied the study inclusion and exclusion criteria. A patient may have had more than one reason for non‐eligibility. Percentages are based on patients entered in the CRF. ^‡^Includes patients with unknown study period. ^§^Percentages are calculated over the total number of patients included by specialists. Note: The sum of patients in the pre‐RMM and post‐RMM periods will not always add up to the total because the total column also includes patients who could not be classified into a study period. Patients without informed consent have not been included in this figure because their data have been removed from the database

Data of 1326 patients were entered in the Case Report Form (CRF) (Figure 2). From the 1217 (91.8%) eligible patients, 711 were included in pre‐RMM period and 506 in post‐RMM period. Patient characteristics are summarized in Table 2. Patients in both study periods were comparable except for history of HF and of hypertension, which were more frequent in patients with data collected during the post‐RMM period, and history of sinus bradycardia was less frequent in the post‐RMM period. Mean (standard deviation) total treatment duration during the 6‐month follow‐up period was similar for both periods (18.0 [9.41] weeks in the pre‐RMM period and 17.0 [9.70] in the post‐RMM period).

**Table 2:** Patient characteristics

Variable	Pre‐RMM (N = 711)	Post‐RMM (N = 506)	*P* value
Sex (n [%])			
Male	442 (62.2)	318 (62.8)	.8213
Female	269 (37.8)	188 (37.2)	
Age at ivabradine initiation (y), (n [%])			
18‐44	21 (3.0)	11 (2.2)	.5856
45‐64	226 (31.8)	178 (35.2)	
65‐74	217 (30.5)	162 (32.0)	
75‐84	195 (27.4)	119 (23.5)	
≥85	52 (7.3)	36 (7.1)	
Under 75	464 (65.3)	351 (69.4)	.2551
75 and over	247 (34.7)	155 (30.6)	
Smoking status (n [%])			
Current smoker	91 (12.8)	70 (13.8)	.7993
Past smoker	267 (37.6)	192 (37.9)	
Nonsmoker	283 (39.8)	191 (37.7)	
Unknown	70 (9.8)	53 (10.5)	
Medical history (n [%])			
Hypertension	541 (76.1)	410 (81.0)	.0313
Hyperlipidaemia	488 (68.6)	329 (65.0)	.2142
Coronary angioplasty	310 (43.6)	229 (45.3)	.8224
Diabetes mellitus (Type 1 or 2)	280 (39.4)	199 (39.3)	.8007
Overweight or obese	242 (34.0)	196 (38.7)	.0983
Peripheral vascular disease	142 (20.0)	113 (22.3)	.4943
Coronary artery bypass	135 (19.0)	84 (16.6)	.2573
Heart failure	122 (17.2)	147 (29.1)	<.0001
Sinus bradycardia	33 (4.6)	10 (2.0)	.0146
Other conduction disorders	32 (4.5)	27 (5.3)	.4631
Atrial fibrillation or atrial flutter—Paroxysmal	29 (4.1)	29 (5.7)	.1623
Atrioventricular block	26 (3.7)	10 (2.0)	.1804
Other supraventricular arrhythmias	22 (3.1)	10 (2.0)	.2428
Ventricular tachycardia—Not sustained	21 (3.0)	16 (3.2)	.7949
Atrial fibrillation or atrial flutter—Persistent	13 (1.8)	6 (1.2)	.3008
Ventricular tachycardia— Sustained	12 (1.7)	6 (1.2)	.3881
Other ventricular arrhythmias	11 (1.5)	8 (1.6)	.9370
QT interval prolongation	5 (0.7)	5 (1.0)	.5789
Ivabradine initiation status (n [%])			
Initiated in the participating practice/clinic	553 (77.8)	385 (76.1)	
Initiated outside the participating practice/clinic	158 (22.2)	121 (23.9)	
Status of subsequent prescription(s) (n [%])			
Written by physician in the practice/clinic	400 (56.3)	268 (53.0)	
Written by physician outside of the practice/clinic	168 (23.6)	148 (29.2)	
Physician has no information on subsequent prescription(s)	143 (20.1)	90 (17.8)	

Abbreviation: RMM, risk‐minimization measures.

#### 3.1.1 Ivabradine dosing at treatment initiation and during follow‐up

Overall, 660 patients (92.8%) and 475 patients (94.1%) in the pre‐RMM and post‐RMM periods had initial ivabradine doses less than or equal to 5‐mg bid, in line with the SmPC. Results were similar regardless of age (92.0% of patients aged <75 years in the pre‐RMM and 92.9% in the post‐RMM periods and 94.4% of patients aged ≥75 years in the pre‐RMM and 96.1% in the post‐RMM periods had initial ivabradine doses of ≤5‐mg bid).

In the post‐RMM period, the percentages of patients prescribed the lowest initial dose of ivabradine (≤2.5‐mg bid) increased compared with the pre‐RMM period. This increase was greater for patients aged greater than or equal to 75 years (29.6% [n = 73] in the pre‐RMM period to 36.1% [n = 56] in the post‐RMM period) relative to those aged less than 75 years (25.0% [n = 116] in the pre‐RMM period to 30.2% [n = 106] in the post‐RMM period).

During follow‐up, there were no ivabradine prescriptions that exceeded 7.5‐mg bid in either pre‐ or post‐RMM periods, in line with SmPC.

#### 3.1.2 Concomitant medication use

The proportion of patients with no concomitant use of verapamil or diltiazem increased in post‐RMM period (96.1% and 99.2% in the pre‐RMM and post‐RMM periods, difference: 3.2; 95% CI, 1.3‐5.0; *P* value = .0007).

#### 3.1.3 HR at initiation

The proportion of patients with HR ≥ 70 bpm at treatment initiation increased in post‐RMM period compared with pre‐RMM period (521 patients [79.4%] in the pre‐RMM period and 396 patients [85.2%] in the post‐RMM period; *P* value = .0141).

### 3.2 Overall patterns of use pre‐ and post‐RMM

The overall proportion of patients treated with ivabradine according to the composite of the four criteria of SmPC increased in the post‐RMM period (70.6% in the pre‐RMM period and 78.4% in the post‐RMM period, difference: 7.8; 95% CI, 2.5‐12.9; *P* value = .0035) (Table 3). As per country, the proportion of patients treated with ivabradine according to the composite of the four criteria of SmPC increased in the post‐RMM study period compared with pre‐RMM period in France, Germany, Spain, and the UK but not in Italy mainly because of a lower‐HR threshold adherence (84.2% in pre‐RMM vs 79.8% in post‐RMM). Similar increase in compliance to the RMM was observed in both GPs and specialists (Table 4 **)**.

**Table 3:** Compliance with RMMs: Overall pattern of ivabradine prescribing

Criteria	Pre‐RMM (N = 711) (n [%])	Post‐RMM (N = 506) (n [%])	Difference (95% CIs)	*P* value
Heart rate				
Heart rate at treatment initiation ≥70 bpm^a^	521 (79.4)	396 (85.2)	5.7 (1.0‐10.3)	.0141
Unknown/missing (%)	55 (7.7)	41 (8.1)		
SmPC dose				
No ivabradine dose higher than the SmPC doses at treatment initiation and during follow‐up	660 (92.8)	475 (94.1)	1.2 (−1.8 to 4.1)	.3957
Unknown/missing (%)	0 (0)	1 (0.2)		
No ivabradine dose higher than the SmPC doses at treatment initiation	660 (92.8)	475 (94.1)	1.2 (−1.8 to 4.1)	0.3957
Unknown/missing (%)	0 (0)	1 (0.2)		
No ivabradine dose higher than the SmPC doses during follow‐up (among patients with renewals data)^b^	382 (100)	276 (100)	0.0 (−1.2 to 1.7)	NC
Patients with renewals data	382	276		
Missing dose among renewals recorded (%)	0 (0)	0 (0)		
Verapamil/diltiazem use				
No concomitant use of verapamil or diltiazem at ivabradine treatment initiation and during follow‐up	683 (96.1)	502 (99.2)	3.2 (1.3‐5.0)	.0007
Unknown/missing (%)	0 (0)	0 (0)		
Treated according to current SmPC^c^ (four criteria)	466 (70.6)	366 (78.4)	7.8 (2.5‐12.9)	.0035
Unknown/missing (%)	51 (7.2)	39 (7.7)		

Abbreviation: bpm, beats per minute; CI, confidence interval; NC, not calculated; RMM, risk minimization measures; SmPC, summary of product characteristics.

a Based on the latest heart rate measurement available prior to or on ivabradine initiation date. If multiple values are available for the same date, this criterion is satisfied if the mean is greater than or equal to 70 bpm.

b Patients with recorded renewals that had missing dose are classified as noncompliant.

Corresponds to patients prescribed ivabradine according to the heart rate recommendation, no doses higher than the SmPC doses at treatment initiation and during follow‐up (if available) and no concomitant use of verapamil or diltiazem during the study period. Patients with missing data for a given criterion but who failed to satisfy another criterion are classified as noncompliant (and not counted in the missing data row).

**Table 4:** Compliance with RMMs: Overall pattern of ivabradine prescribing by country and by specialty

		Treated According to the Four Criteria of Current SmPC^a^	
		Pre‐RMM	Post‐RMM
Country			
France	n patients/N (%)	150/218 (68.8)	69/94 (73.4)
	Unknown/missing	5	5
Germany	n patients/N (%)	112/143 (78.3)	156/187 (83.4)
	Unknown/missing	3	1
Italy	n patients/N (%)	108/134 (80.6)	71/95 (74.7)
	Unknown/missing	20	29
Spain	n patients/N (%)	65/105 (61.9)	33/46 (71.7)
	Unknown/missing	8	2
UK	n patients/N (%)	31/60 (51.7)	37/45 (82.2)
	Unknown/missing	15	2
Specialty			
GPs	n patients/N (%)	114/169 (67.5)	123/153 (80.4)
	Unknown/missing	18	7
Specialists	n patients/N (%)	352/491 (71.7)	243/314 (77.4)
	Unknown/missing	33	32

*Note*. The denominator is patients who reported information for each of the criteria (patients with no subsequent prescriptions are included in the denominator).

Abbreviations: GP, general practitioner; N, total number of patients in the strata; RMM, risk minimization measures; SmPC, summary of product characteristics.

a Corresponds to patients prescribed ivabradine according to the heart rate recommendation, no doses higher than the SmPC doses at treatment initiation and during follow‐up (if available) and no concomitant use of verapamil or diltiazem during the study period.

### 3.3 Sensitivity analysis

Except one patient at initiation in post‐RMM period, doses (at initiation and during follow‐up) and concomitant use with verapamil and diltiazem were completely reported in the patients' chart in both study periods. The percentage of missing key data was higher for HR at initiation (55 patients [7.7%] and 41 patients [8.1%] in pre‐ and post‐RMM periods, respectively) than for the other criteria. The increase in compliance with the new RMMs remained significant when patients with missing data were included in the denominator (*P* value = .0121).

The sensitivity analysis taking into account the physicians' initiator status showed a lower proportion of missing data on HR at initiation when the participating physician was the initiator (4.2% and 3.4% in the pre‐RMM and post‐RMM periods, respectively) than when the participating physician was the subsequent prescriber (20.3% and 23.1%, respectively). The compliance with the four criteria increased between the pre‐ and post‐RMM periods among patients for whom the initiator (ie, who initially prescribed ivabradine) was the participating physician (from 70.4% to 80.2%). No increase was observed in patients recruited by subsequent prescribers.

## 4 DISCUSSION

The overall objective of this PASS was to assess in five European countries how ivabradine is used in patients with chronic stable angina pectoris in routine clinical practice and to evaluate the compliance with the new RMMs. Patients were identified across a variety of physician specialties, including specialists from private and hospital settings, as well as GPs. Overall, the RMMs were well implemented across the participating countries and among both specialists and GPs.

One of the specific study aims was to check that appropriate ivabradine doses were prescribed. These recommended doses had not changed following the benefit‐risk assessment, explaining the compliance being already high in the pre‐RMM period. Absence of dosing higher than 7.5‐mg bid in both periods is also reassuring compared with the SIGNIFY^2^ maintenance dose of 10‐mg bid. Similarly, concomitant use of ivabradine with verapamil or diltiazem, already not recommended before RMM, rarely occurred in both periods. Regarding HR, the threshold in patients with angina changed from greater than 60 bpm to greater than or equal to 70 bpm with RMM implementation. Although 79.4% of patients had a HR ≥ 70 bpm in the pre‐RMM period, this proportion increased significantly post‐RMM.

This study aimed to ensure selection of a diverse and generally representative physicians' sample and their treated patients. However, as for most studies conducted for regulatory reasons,^6^ this study faced a very low interest rate among physicians and recruitment challenges. Having a total of 68 active physicians across the five countries needs to be considered when interpreting the results. The study was not powered to assess compliance with RMMs at country and specialty levels. This is particularly true for Italian GPs and UK specialists (one and two active physicians, respectively). The assessment of any potential difference between participating and nonparticipating physicians was not possible because data on nonparticipating physicians were very scarce. No robust conclusion could be drawn on representativeness of participating physicians compared with the general medical population. However, physicians' sampling was done based on very large source lists, iteratively through six waves of recruitment. A total of 60 675 physicians were invited to participate in the study of whom 522 (0.86%) were interested. This low rate was driven by the strategy of mass mailing, chosen to optimize the absolute number of interested sites and to meet regulatory timelines. Given that invited physicians were informed of the study objectives and that physician participation was voluntary, there was potential for bias towards more participation of physicians who prescribed ivabradine as recommended in the SmPC. However, physicians' recruitment activities started after the post‐RMM period was over, ensuring that prescriptions issued during this period were not influenced by physicians' awareness of the study objectives.

The initial target of 600 patients per period was not reached for the post‐RMM period, which was shorter than the pre‐RMM period; yet, the inclusion of 506 patients was acceptable with a sufficient level of absolute precision to meet the study objective. Patients' characteristics were in line with those usually observed in populations with chronic stable angina pectoris in terms of age and sex distribution and most frequent comorbidities.^7^ HF was significantly more frequent among patients in the post‐RMM period, in line with the new indication of chronic HF granted in 2012.

While low proportions of patients with missing key data were observed during the study, the proportions were higher for HR at initiation (7.7% and 8.1% in the pre‐RMM and post‐RMM periods, respectively). Missingness of data was further impacted by SCM. HR values at initiation were more likely to be missing when the participating physician was a subsequent prescriber. However, it does not imply that the HR measure had not been performed at initiation. Moreover, it can be assumed that patients with missing HR at initiation and included by the subsequent prescriber had HR distribution similar to the one observed among patients with recorded HR and included by the initiator.

Measuring RMMs' effectiveness is an important aspect of a drug benefit‐risk evaluation. This study design is aligned with the guideline on good pharmacovigilance practices (GVP) module XVI,^8^ where a medical chart abstraction is considered as a valuable option to assess clinical knowledge and prescribing behavior and is preferred to surveys or self‐reported data. The pre‐post retrospective study design, widely used among similar PASS and DUS,^9^, ^10^, ^11^, ^12^, ^13^, ^14^ allows for showing the immediate impact of short‐term programs such as RMMs.^15^, ^16^ A review of 29 studies in the European Union electronic Register of Post‐Authorization Studies found that only four studies used retrospective medical files review—others were surveys—and only 10 studies were conducted within a 12–18‐month timeframe after RMM implementation.^17^ The main strength of this PASS remains its conclusiveness on the effectiveness of RMMs on the prescribing of ivabradine since EU marketing authorization. A review of studies registered in EU PAS Register showed that only half of the effectiveness indicators were reported as successful and conclusive.^18^ In addition, the current study did examine the compliance with each individual component of the RMMs and the composite.

Overall, although most patients were already prescribed ivabradine according to RMMs, the study was able to show a significant improvement in compliance to these RMMs in the post‐RMM period.

## 5 CONCLUSION

Overall, the study results show that the RMMs for ivabradine were well implemented across the participating European countries. Ivabradine prescribing patterns have significantly changed to be in line with newly implemented RMMs and the updated SmPC, confirming a favorable benefit‐risk balance of ivabradine in chronic stable angina pectoris and maintaining the ivabradine EU marketing authorization.

## ETHICS STATEMENT

All required national or local ethical committees approvals were obtained.

## CONFLICT OF INTEREST

Linda Salem, Nicolas Deltour, and Emmanuelle Jacquot are employees of Institut de Recherches Internationales Servier. Alexandre Malouvier and Jon Blatchford are employees of PRA Health Sciences, the Contract Research Organization to which the research project was contracted by Servier, the company that owns ivabradine. E Rivero‐Ferrer works for RTI Health Solutions, a business unit of RTI International, which has been compensated for work done for this research. RTI International is an independent, nonprofit research organization that conducts work for government, public, and private organizations, including pharmaceutical companies.

## AUTHORS CONTRIBUTION

Authors E.J., N.D., and E.R.F. planned the study. E.J., N.D., L.S., and E.R.F. conducted feasibility evaluation and drafted the study protocol. E.J., N.D., and L.S. made contributions to the final design and final approved version of the protocol. J.B. undertook the statistical analysis of the study. J.B. completed the analysis, and all authors contributed in the interpretation of the results. All authors contributed to the writing of the first draft of the manuscript. All authors contributed to and have approved the final manuscript.
